# Aspects of medical migration with particular reference to the United Kingdom and the Netherlands

**DOI:** 10.1186/1478-4491-12-59

**Published:** 2014-10-14

**Authors:** Paul GP Herfs

**Affiliations:** European Research Centre on Migration and Ethnic Relations (ERCOMER), Utrecht University, Utrecht, the Netherlands

**Keywords:** International medical graduates, Migrating doctors, Brain drain, Language requirements for foreign doctors

## Abstract

**Background:**

In most countries of the European Economic Area (EEA), there is no large-scale migration of medical graduates with diplomas obtained outside the EEA, which are international medical graduates (IMGs). In the United Kingdom however, health care is in part dependent on the influx of IMGs. In 2005, of all the doctors practising in the UK, 31% were educated outside the country. In most EEA-countries, health care is not dependent on the influx of IMGs.

The aim of this study is to present data relating to the changes in IMG migration in the UK since the extension of the European Union in May 2004. In addition, data are presented on IMG migration in the Netherlands. These migration flows show that migration patterns differ strongly within these two EU-countries.

**Method:**

This study makes use of registration data on migrating doctors from the General Medical Council (GMC) in the UK and from the Dutch Department of Health. Moreover, data on the ratio of medical doctors in relation to a country’s population were extracted from the World Health Organization (WHO).

**Results:**

The influx of IMGs in the UK has changed in recent years due to the extension of the European Union in 2004, the expansion of UK medical schools and changes in the policy towards non-EEA doctors.

The influx of IMGs in the Netherlands is described in detail. In the Netherlands, many IMGs come from Afghanistan, Iraq and Surinam.

**Discussion and conclusions:**

There are clear differences between IMG immigration in the UK and in the Netherlands. In the UK, the National Health Service continues to be very reliant on immigration to fill shortage posts, whereas the number of immigrant doctors working in the Netherlands is much smaller. Both the UK and the Netherlands’ regulatory bodies have shared great concerns about the linguistic and communication skills of both EEA and non-EEA doctors seeking to work in these countries. IMG migration is a global and intricate problem. The source countries, not only those where English is the first or second language, experience massive IMG migration flows.

## Background: IMG influx in the United Kingdom and the Netherlands

Over the last two decades, international medical graduates (IMGs) with diplomas obtained in developing countries have been moving to countries of the European Economic Area (EEA). Most EEA-countries do not actively recruit IMGs; the numbers of IMGs working in EEA-countries are small, with the exception of the UK. In Australia, Canada, the United Kingdom and the United States of America however, migration of IMGs takes place on a large scale. The expression *large-scale IMG-migration* is used when at least 20% of the doctors at work in the host country have been trained elsewhere.

The large-scale IMG-migration movements in Australia, Canada, the UK and the USA have been categorized as a *medical carousel* [1]. For Eastwood *et al*. [1] this carousel starts in developing countries where doctors seek better educational opportunities, more favourable working conditions and higher salaries. As a result a significant proportion of qualified doctors leave their countries of origin. This has been seen in countries such as Tanzania, Kenya and Nigeria where doctors have moved post qualification to settle and work in South Africa. Medical doctors from South Africa also leave their country of training due to an increasing lack of safety and often seek to settle in the United Kingdom. From the UK, many medical doctors will look to migrate on to Canada and the USA in addition to onward migration of Canadian doctors moving south of the border to the USA. The net result is that the number of doctors decreases, especially in African countries, with serious detrimental effects for those countries [2–5]. Moreover, these doctors do not return to their native country after obtaining a full licence in the country they migrated to.

In the UK, solutions have been developed to counter the effects of this medical carousel. Four new medical schools have been established to shift the emphasis to producing more UK graduates to staff the National Health Service (NHS). In parallel to this the UK has also changed its policy towards IMGs making it more prohibitive for those medical graduates that have trained outside the EEA to join national training programmes. The removal of the open door policy and introduction of work permits in April 2006 signalled a change in recruitment policy where non-EEA doctors are only sought if there are clear vacancies in training posts [6]. While this policy was new within the UK, it had been standard practice in many other EEA-countries.

These visa changes were challenged by many IMGs (mostly from India and Pakistan) who were already in the UK seeking employment. However, in February 2007, the High Court decided that the new guidance was lawful. There has been strong criticism of this cutting of the training route for non-EEA doctors [7]. A survey study conducted in 2007 by George *et al*. [8] showed that of those 1,617 IMGs surveyed, 76% cited ‘further training’ as their main reason for migration to the UK.

In contrast to the UK, most IMGs in the Netherlands are either refugees or individuals who want to join their spouses/partners. A study by Herfs and Haalboom [9] showed that in 2002 the majority (71%) of IMGs starting their additional training at medical schools had a refugee status, while in 2005 the majority (57%) had a status as spouses of Dutch citizens. These changes in IMGs backgrounds are the result of policy changes in Dutch immigration law. One such example is the Aliens Law [10] that came into force in 2001 and aimed at sharpening the immigration laws and at making the regulations stricter for asylum seekers. Generally, the external recruitment of medical doctors hardly occurs in the Netherlands. However, there was one exception: in the period 1999 to 2002 medical doctors were actively recruited in South Africa due to short-term shortages in Dutch hospitals. In February 2007, a Dutch recruiter of South African doctors was sentenced to 8 months in prison due to a breach of immigration regulations during this period [11].

The *medical carousel* model put forward by Eastwood *et al*. [1] is not a phenomenon that occurs in EEA-countries with small-scale IMG-migration. As a result, some source countries may end up with a low ratio of doctors if many of their doctors leave in order to settle permanently in other countries. Even though the overall number of migrating doctors to EEA-countries may be small, for these source countries the impact of the migration of their medical workforce is significant.

## Methods

In this study, the term *ratio of doctors* is used to represent the number of doctors per 100,000 citizens of a country. The aim of this study was to compare these ratios in the Netherlands and other western countries with that of source countries, whose trained medical doctors leave to work elsewhere. To that end, data on the IMG influx were studied from the Dutch Department of Health as well as from the World Health Organization (WHO) [12]. The WHO has records on health care workers in all the countries in the world. Based on these WHO data, estimations were made about possible shortages of doctors in the countries that are compared in this study. Of course, we are aware of the fact that these data are only indications for chances of IMGs on the labour market. The lower the ratio, the better the chances for IMGs to find work in health care in that country. For the UK, registration data on IMGs were gathered from the General Medical Council.^a^

## Pull factors for IMGs to choose for the UK and the Netherlands

IMGs often have very different reasons to migrate to the United Kingdom or the Netherlands. Medical migration to and from the United Kingdom is a well-established phenomenon [13]. From as early as 1599, British medical doctors migrated overseas to India, and in the eighteenth century British doctors started to train Indians in western medicine. Two-sided medical migration processes are a direct result of the British colonial history. While the Netherlands has its own colonial history, medical migration based on these colonial ties was never as extensive as in the UK. Table 1 presents the differences in migration pull factors for IMGs for the UK or the Netherlands.

**Table 1:** **Pull factors for international medical graduates (IMGs) and number of accepted asylum seekers in 2012**

Pull factors for IMGs	United Kingdom	Netherlands
Long-lasting history of medical migration	yes	no
Same language as in country of origin	yes	no
Quick assessment procedure	yes, since 1975	yes, since 2005
Medical schools experienced in additional training of IMGs	yes	no
After medical study: working permit necessary for non-EEA doctors	yes, since 2006	yes, unless permanent visa
After medical study: possibilities for consultant training position	yes; until 2006	yes, but only for locally trained IMGs
Medical staff in hospitals is international	yes	no
Health care is dependent on IMGs	yes	no
Better remuneration than in country of origin	yes	yes
Primary reason to migrate: further medical education	yes	no
Number of accepted asylum seekers in 2012	14,570	5,920

## Results and discussion

The total numbers of IMGs seeking recognition in the Netherlands are set out in Table 2. In the year 2000, a total of 257 IMGs, originating from 51 countries, requested recognition.^b^ Relatively large numbers came from South Africa (33), Afghanistan (23), Iraq (17), the former USSR (26), Poland (20), Romania (10) and Surinam (8). Since 2003, in order to protect the privacy of the applicants, the Dutch Department of Health has ceased publishing the nationalities of IMGs. Unfortunately, this makes more detailed analysis and cross-country comparisons much more difficult.

**Table 2:** **Numbers of international medical graduates (IMGs) asking recognition at the Dutch Department of Health**

Year	Number of International Medical Graduates
1995	292
1996	362
1997	357
1998	337
1999	274
2000	257
2001	no annual report available
2002	303
2003	no annual report available
2004	no annual report available

Source: annual reports Department of Health.

An international comparison showed that the number of migrating IMGs to the UK was higher than the number of migrating IMGs to other EEA-countries [14]. In 2003, the number of IMGs seeking registration to practise in the UK amounted to a total of 13,967 (see Table 3). This number represents 10.5% of all UK-doctors (see Table 4). This is in stark contrast with the 303 IMGs who applied for recognition in the Netherlands in 2002. If all of these doctors had been recognized this would have represented only 0.5% of all Dutch doctors.

**Table 3:** **International medical graduate** (**IMG)-registration figures from the General Medical Council (2002 to 2007)**

Country	2002	2003	2004	2005	2006	2007
Bulgaria	14	14	37	48	27	92
Czech Republic	28	31	228	270	137	110
Cyprus	0	0	0	0	0	0
Estonia	1	1	12	25	12	2
Hungary	21	41	189	320	228	194
Latvia	3	4	21	52	25	11
Lithuania	1	2	38	129	65	31
Malta	17	18	32	39	55	40
Poland	21	19	498	744	532	339
Slovakia	3	5	49	85	76	42
Slovenia	0	3	3	5	7	3
Romania	31	37	65	74	87	175
Total international applicants	6,828	13,967	10,407	9,934	6,159	5,055
United Kingdom	4,398	4,734	4,333	5,164	5,620	6,133
Total	11,226	18,701	14,740	15,098	11,779	11,188

Source: General Medical Council (2007).

**Table 4:** **Number of doctors per 100,000 citizens in 18 European Economic Area (EEA)-countries**

Country	Total number of doctors	Number of doctors per 100,000 citizens
Austria	27,413	338
Belgium	46,268	449
Denmark	15,653	293
Finland	16,446	316
France	203,487	337
Germany	277,885	337
Greece	47,944	438
Iceland	1,056	362
Ireland	11,141	279
Italy	241,000	420
Luxembourg	1,206	266
Netherlands	50,854	315
Norway	14,200	313
Portugal	34,440	342
Spain	135,300	330
Sweden	29,122	328
Switzerland	25,921	361
United Kingdom	133,641	230

Source: WHO; World Health Statistics; 2007.

The same pattern can be found when looking at the migration of IMGs into Scandinavian countries, Austria and Belgium (Flanders only). In these EEA-countries, the countries of origin of the IMGs appeared to be very similar [14]. All countries received requests for recognition from IMGs originating from Afghanistan, Iraq, Russia and other former Eastern *bloc* countries. By contrast, in the UK the majority of requests came from IMGs originating from countries belonging to the British Commonwealth [14].

The UK pattern of large influxes of IMGs from Commonwealth countries has changed since the UK Immigration Act was changed with the abolition of Section 19 in 2003. Section 19 gave *de facto* recognition of overseas qualifications which allowed doctors with qualifications obtained in the Commonwealth countries to obtain full registration in the same way as those who held a UK primary medical qualification. In other words, doctors from Commonwealth countries were not obliged to take an entrance examination.

Section 19 was terminated in 2003 which might help to explain the larger number of IMGs seeking registration in that year. The abolition of Section 19 and expansion of the European Union have led to a greater variety in the source countries for UK IMGs and a growth in the number of IMGs who had been resident in other European countries. In 2014, the General Medical Council was again requested to present data on IMG registration. Table 5 presents the data over the period 2008 to 2013, provided by the GMC.

**Table 5:** **International medical graduate** (**IMG)-registration figures from the General Medical Council (2008 to 2013)**

Country	2008	2009	2010	2011	2012	2013	Total
Bulgaria	120	146	166	139	133	94	798
Czech Republic	93	99	116	141	138	124	711
Estonia	9	4	9	6	17	5	50
Hungary	169	172	207	185	188	125	1,046
Latvia	13	49	59	25	27	22	195
Lithuania	28	33	71	45	42	38	257
Malta	60	35	20	23	42	39	219
Poland	203	196	157	115	161	164	996
Romania	233	254	677	449	292	276	2,181
Slovakia	37	38	40	40	53	44	252
Slovenia	5	5	7	3	22	10	52
Non-EEA	2,826	2,579	2,959	2,437	2,222	2,379	15,402

Source: General Medical Council (2014).

In the past four years, more EEA-doctors than non-EEA-doctors have been successful in obtaining registration with the UK General Medical Council. This might be, in part, a consequence of the expansion of the EU and an increasingly hostile approach to both EEA and non-EEA-migrants. While the latter are subject to visa regulations,^c^ the former can enjoy the patronage of freedom of movement within the European Union.

Since 2010, the influx of EEA doctors has increased with larger numbers migrating to the UK from Bulgaria, Hungary, Poland and Romania. However, since the outbreak of the financial crisis, IMGs from ‘older’ EU countries have also found their way to the UK. Since 2008, 2,120 Greek, 2,445 Italian and 1,078 Spanish medical doctors have registered with the GMC. These figures show that many doctors will find jobs in health care in the UK, whether their origins lie in the old or in the new EU countries.

More than 20% of medical doctors in countries with large-scale IMG-migration, that is Australia, Canada, the UK and the USA, have foreign medical degrees [15, 16]. This may imply that a shortage of medical personnel is effectively replenished with IMGs in these countries. In most EEA-countries, with the exception of the UK, there is not such an obvious shortage. The data in Table 4 show that the ratio of doctors is lower in the United Kingdom than in other EEA-countries. The average ratio of doctors for these 18 EEA-countries is 336, whereas for the United Kingdom it is 230. The ratios differ in part due the way in which each health system is designed but the number of EEA doctors seeking employment in the UK is perhaps indicative of shortages in doctors that this ratio might suggest.

Table 6 shows the numbers of medical doctors and the ratio of doctors in three G8 (*Group of Eight)* countries and Australia. The numbers for these countries are more comparable to the United Kingdom than to the numbers in the other EEA-countries.

**Table 6:** **Numbers of doctors per 100,000 citizens in 4 non-European Economic Area (EEA) countries**

Countries	Total number of doctors	Number of doctors per 100,000 citizens
Canada	66,583	214
USA	730,801	256
Japan	251,889	198
Australia	47,875	247

Source: WHO; World Health Statistics; 2007.

Table 7 lists the data with regard to the source countries. This includes the numbers of remaining medical doctors and the ratio of doctors.

**Table 7:** **Numbers of doctors per 100,000 citizens in countries of origin of international medical graduates (IMGs) settling in the Netherlands**

Country	Total number of doctors	Number of doctors per 100,000 citizens
Afghanistan	4,104	19
China	1,364,000	106
Iran	60,791	87
Iraq	17,022	66
Russian Federation	609,043	425
Ukraine	143,202	295
Indonesia	29,499	13
Surinam	191	45

Source: WHO; World Health Statistics; 2007.

Afghanistan is a country with a very low ratio of doctors; for the past ten years, this country has been confronted with a large emigration of medical doctors to western countries fleeing war and persecution [14]. According to the national refugee organization in the Netherlands, 2,552,208 Afghani refugees have left their country in 2013 [17]. This means that one quarter of all refugees worldwide is of Afghan origin. Similar large-scale emigrations of medical doctors have also occurred in Iraq and, more recently, Syria. The Netherlands and other Western European countries granted many Afghan and Iraqi refugees permanent residency and enabled the refugee doctors to obtain doctors’ licences. These relatively large flows of Afghan and Iraqi doctors may have serious consequences for the health care in their native countries; this can be deduced from the data in Table 7. In countries where the ratio of doctors was already low before the start of emigration, even the migration of a limited number of medical doctors can have severe consequences. After the normalization of living conditions in these countries, refugees might consider returning to their country of origin. However, as yet no signs of normalization of living conditions can be noticed in these two countries and there is no demonstrable return of doctors despite requests from national governments for those doctors that had fled to return home to support the understaffed health service. In 2008, the Iraqi government made such a request to those Iraqi doctors that had fled the country during the war to return home.

In the Russian Federation and Ukraine there is a relatively high ratio of doctors; therefore, it is unlikely that the emigration of medical doctors will damage health care in these countries to the same extent as it has in countries such as Afghanistan and Iraq. The Netherlands has a colonial history with Indonesia and Surinam. As a result, data concerning the numbers of medical doctors in these two countries have been included in Table 7. Health care provision in these two countries may also be damaged due to the fact that a certain percentage of the total number of medical doctors is leaving Indonesia and Surinam in order to settle in the Netherlands. In particular, in Surinam, the consequences of migrating IMGs could be severe.

On 1 May 2004, the European Union was extended with 10 additional European countries, and on 1 January 2007, Bulgaria and Romania joined the EU. The numbers of medical doctors and the ratio of doctors in these countries are represented in Table 8.

**Table 8:** **Numbers of doctors per 100,000 citizens in 12 new European Union (EU)-countries**

Country	Total number of doctors	Number of doctors per 100,000 citizens
Bulgaria^a^	28,128	56
Cyprus	1,864	234
Czech Republic	35,960	351
Estonia	6,118	448
Latvia	6,940	301
Lithuania	13,682	397
Hungary	32,877	333
Malta	1,254	318
Poland	95,272	247
Romania	42,538	190
Slovakia	17,172	318
Slovenia	4,475	225

^a^Bulgaria and Romania joined the European Union on 1 January 2007. The other 10 countries joined the EU on 1 May 2004.

Source: WHO; World Health Statistics; 2007.

In these new EU or EEA-countries, the average number of doctors is 310 per 100,000 inhabitants, and this ratio is comparable to that of the old European Union countries (which is 336 on average). Medical doctors from the new countries (except Bulgaria and Romania) who started their medical education after 1 May 2004 will be covered by a European Directive [18] regarding the recognition of professional qualifications. This implies that after graduating as medical doctors, they can apply successfully for the recognition of their degree in any EU-country. For medical doctors who graduated before the extension of the European Union, successful recognition is not guaranteed. The pending ‘easy’ recognition leads to the fear in some new European Union countries, for example, Hungary [19] and Poland [20], that large numbers of medical doctors will eventually leave their country to settle in European countries with higher social standards and, especially, higher salaries.

In 2006, groups of medical doctors and nurses in Poland have already demonstrated for higher salaries, threatening to leave the country if their terms were not met. In the past two years, the number of medical doctors in Romania has decreased from 20,000 to 14,000. These doctors have left Romania because of the low wages and the excessive corruption in health care [21]. Recently, the Romanian Labour Minister warned against the negative effects of the emigration of highly skilled workers, in particular doctors [22]. The effect of this warning is, as yet, unknown.

## Conclusions

Overall, seven conclusions can be drawn.

1. The composition of the groups of IMGs in Western European countries is fairly uniform, with the exception of the UK, where many IMGs originate from Commonwealth countries. These groups consist of refugees and of people seeking to be with their spouses/partners. IMG migration to the UK decreased after 2003 (when Section 19 was abolished, which provided recognition of overseas qualifications). After the extension of the EU in 2004, there has been a strong increase in the influx of IMGs originating from the new EU-countries, with Polish and Hungarian doctors in particular coming to the UK in great numbers. In 2005, 744 Polish doctors and 320 Hungarian doctors went overseas. However, by 2007 these numbers had decreased to 339 and 194, respectively (see Table 3).

2. In some of the new European Union countries such as Hungary and Poland, it was feared that large numbers of medical doctors would leave these countries after they had joined the European Union, as recognition would be easily arranged by an EU-directive. This fear was not misplaced. Romania and Croatia, the youngest EU-member state, also fear that their medical doctors will leave their countries.

3. In most European countries there is no large-scale IMG migration, which is in sharp contrast with Australia, Canada, the UK and the USA. These 4 countries have historically depended on the influx of IMGs with more than 20% of the practising medical doctors in these countries holding foreign medical degrees. As European countries only have a small-scale IMG-influx, health care is not dependent on IMGs. After changing the migration rules for non-EEA doctors the UK became less attractive for this group. The gap on the labour market for foreign doctors was immediately filled up by IMGs from Romania, Hungary, Poland, Bulgaria and the Czech Republic. Since October 2008, the influx of Eastern European citizens in the UK has been changing rapidly due to the global financial crisis.

4. There are remarkable differences between the IMGs going to the UK and those going to the Netherlands. In the UK, foreign-trained medical professionals are necessary, although measures have been taken to reduce the numbers of IMGs as the expansion of medical schools has resulted in the production of more UK trained medical graduates facing harsh competition for training posts. For many years the UK government facilitated the access of IMGs to the medical profession needed to fill vacancy posts. After all, this lead to the situation in which highly skilled workers from abroad, whose education was paid for by another government, intended to work for the British NHS. In the Netherlands, however, access is much more difficult. IMGs’ educational backgrounds are often evaluated as inferior to the Dutch medical education. Moreover, IMGs seeking recognition in the Netherlands do not master the Dutch language, which is in shrill contrast to many IMGs emigrating to the UK. There are severe language requirements for IMGs in the Netherlands. Since the introduction of the new assessment procedure, many IMGs have failed to obtain a possibility to meet Dutch medical standards [23]. As a result these IMGs will not be able to work as medical doctors in the Netherlands.

5. The Netherlands and the UK have become more demanding concerning the mastery of the languages by migrating doctors. In the UK English language tests for all non-UK graduates (non-EEA and EEA doctors) will be standard practice onwards the summer of 2014 [24].

6. The migration of IMGs is probably an unstoppable phenomenon; it creates problems for those source countries with insufficient numbers of health professionals for their own health system. The migration mainly takes place from poor to rich countries. This applies to countries importing IMGs on a large scale as well as on a small scale. However, IMGs have different motives for settling in countries with a large-scale import than for settling in countries with a small-scale import. The latter countries are usually selected by IMGs who are either refugees or people wanting to join their spouses or partners. Mostly, these IMGs are not familiar with the language of the country they want to settle in; they are not primarily seeking residency as medical doctors, but as husbands, wives or partners or fleeing persecution.

7. IMG migration is a global and intricate issue. The source countries, not only those where English is the first or second language, are confronted with massive IMGs migration outflows [25]. The WHO [26] should again try to encourage a change of policy in the host countries concerning the import of IMGs and should stimulate countries to enter into agreements to reverse this brain drain.

## Endnotes

^a^The General Medical Council provided the following statistics (Herfs *et al.*, 2007):

2002: 4,456 overseas qualified doctors registered with the GMC

2003: 9,336 overseas qualified doctors registered with the GMC

2004: 686 overseas qualified doctors registered with the GMC

^b^Of these 257 doctors, 44 received direct recognition, 97 partial recognition and 116 had to obtain a medical licence via the universities. From these 116 IMGs, 47 (41%) came from countries at war, such as Afghanistan and Iraq. The remaining 69 (59%) were not from countries at war, but merely wished to settle with their spouses or partners. This picture is mirrored in other EEA-countries.

^c^Non-EEA doctors can apply for the Medical Training Initiative scheme. This programme allows international trainees to come to the UK for a maximum of 2 years. IMGs can benefit from the knowledge, skills and techniques offered by the UK NHS and will use the skills they learn to improve the level of patient care in their home countries on their return. UK hospitals benefit from increased workforce capacity and the skills and knowledge that IMGs can bring. Due to shortages in particular specialties, the Medical Training Initiative Scheme can be extended to 4 years for some specialties. For example, the recent extension to 4 years for Indian doctors working in emergency medicine can be seen as a temporary solution to fill vacancy posts in the NHS.

## Abbreviations

EEA: European Economic Area
ERCOMER: European Research Centre on Migration and Ethnic Relations
EU: European Union
GMC: General Medical Council
IMG: International Medical Graduates
NHS: National Health Service
UK: United Kingdom
WHO: World Health Organization.

## References

[1] Eastwood JB, Conroy RE, Naicker S, West PA, Tutt RC, Plange-Rhule J (2005). Loss of health professionals from sub-Saharan Africa: the pivotal role of the UK. Lancet.

[2] Ogowe A (1996). Brain drain: colossal loss of investment for developing countries. Courier ACP-EU.

[3] Ehman AJ, Sullivan P (2001). South Africa appeals to Canada to stop recruiting its MDs. Can Med Assoc J.

[4] Hagopian A, Thompson MJ, Fordyce M, Johnson KE, Hart LG (2004). The migration of physicians from sub-Saharan Africa to the United States of America: measures of the African brain drain. Hum Resour Health.

[5] Pang T, Lansang MA, Haines A (2002). Brain drain and health professionals. Br Med J.

[6] Dyer C (2007). High Court rejects overseas doctors’ challenge of UK work restrictions. Br Med J.

[7] Dyer O (2007). Doctors criticise proposals to cut specialist training for non-EU graduates. Br Med J.

[8] George JT, Rozario KS, Anthony J, Jude EB, McKay GA (2007). Non-European Union doctors in the National Health Service: why, when and how do they come to the United Kingdom of Great Britain and Northern Ireland?. Hum Resour Health.

[9] Herfs PGP, Haalboom JRE (2008). Studievoortgangsproblemen van buitenlandse artsen die instromen in een hoger jaar van de opleiding geneeskunde. [Study problems of IMGs in Dutch medical schools]. Tijdschrift voor Medisch Onderwijs.

[10] Vreemdelingenwet (2000). Immigration Law 2000. Staatsblad: Sdu Uitgevers.

[11] NRC (2007). Bemiddelaar krijgt acht maanden cel. [Recruiter sentenced to 8 months prison].

[12] WHO (2007). World Health Statistics 2007.

[13] Trewby P (2008). International medical graduates: lessons from the past and hopes for the future. Clin Med.

[14] Herfs PGP, Kater L, Haalboom JRE (2007). Non-EEA-doctors in EEA-countries: doctors or cleaners. Med Teach.

[15] Herfs PGP (2009). Buitenlandse artsen in Nederland. [International Medical Graduates in the Netherlands] Dissertation.

[16] Herfs PGP, Kater L, Haalboom JRE, Herfs PGP (2011). Large-scale migration of International Medical Graduates to Australia, Canada, UK and USA. International Medical Graduates in the Netherlands.

[17] Vluchtelingenwerk Nederland (2013). Vluchtelingenwerk feiten en cijfers.

[18] *Directive 2005/36/EC of the European Parliament and of the Council of 7 September 2005 on the recognition of professional qualifications*. Brussels, Belgium; [http://eur-lex.europa.eu/LexUriServ/LexUriServ.do?uri=OJ:L:2005:255:0222:0142:en:PDF]

[19] NRC (2005). Hongaarse artsen massaal op drift. [Hungarian MDs run away].

[20] NRC (2004). Uittocht van Poolse artsen. [Exodus of Polish MDs].

[21] De Kwant L (2014). Uittocht van artsen uit Roemenië. [Exodus of Romanian MDs].

[22] Fontanella-Khan J (2014). Romanian labour minister warns of disastrous skills exodus.

[23] Herfs PGP (2013). De assessment procedure voor buitenlandse artsen: een balans na 7 jaar. [The assessment procedure for IMGs: drawing up a balance after 7 years].

[24] General Medical Council (2014). GMC welcomes support to check language skills of European doctors.

[25] Stillwell B, Diallo K, Zurn P, Dal Poz MR, Adams O, Buchan J (2003). Developing evidence-based ethical policies on the migration of health workers: conceptual and practical challenges. Hum Resour Health.

[26] WHO (2008). The WHO Code of Practice on the International Recruitment of Health Personnel; 2008.

